# Thoracoscopic Closure of Alveolar Pleural Fistula by Application of Pleural Flap and Polymeric Sealant

**DOI:** 10.1111/1759-7714.70156

**Published:** 2025-09-12

**Authors:** Alfonso Fiorelli, Noemi Maria Giorgiano, Angela Iovine, Antonella Tamburrino, Gaetana Messina

**Affiliations:** ^1^ Thoracic Surgery Unit University of Campania “Luigi Vanvitelli” Naples Italy; ^2^ Anesthesiology Unit University of Campania “Luigi Vanvitelli” Naples Italy

## Abstract

Persistent air leaks due to alveolar pleural fistula following lung resection were a frustrating clinical condition for patients and clinicians, associated with a prolonged hospital stay and increased morbidity. Several surgical strategies have been reported over the years for the closure of alveolar fistula, but the best treatment was still debated. Herein, we reported the clinical case of a patient who experienced a persistent air leak due to alveolar‐pleural fistula following thoracoscopic right upper lobectomy for the management of early lung cancer. The fistula was successfully closed by thoracoscopic application of a pleural flap and polymeric sealant. Our new strategy could turn out to be useful for surgeons when standard procedures for management of APF were unfeasible or difficult to perform. Obviously, our impression should be validated by future large studies in a prospective manner.

## Graphical abstract

The lung was not expanded for the presence of air‐leaks. After thoracoscopic closure of the fistula by pleural flap, the air‐leaks ceased, and the lung was fully expanded.

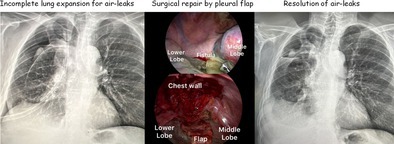

## 1 Background

Persistent air leaks due to alveolar pleural fistula (APF) following lung resection are a frustrating clinical condition for patients and clinicians, associated with a prolonged hospital stay and increased morbidity [1, 2, 3]. The poor patient clinical condition, the underlying lung disease, and the lack of standardized treatments make management difficult. Herein, we report the successful closure of APF by the application of the pleural flap associated with polymeric sealant (Coseal, Baxter Healthcare, Vien, Austria).

## 2 Clinical Case

**FIGURE 1:**
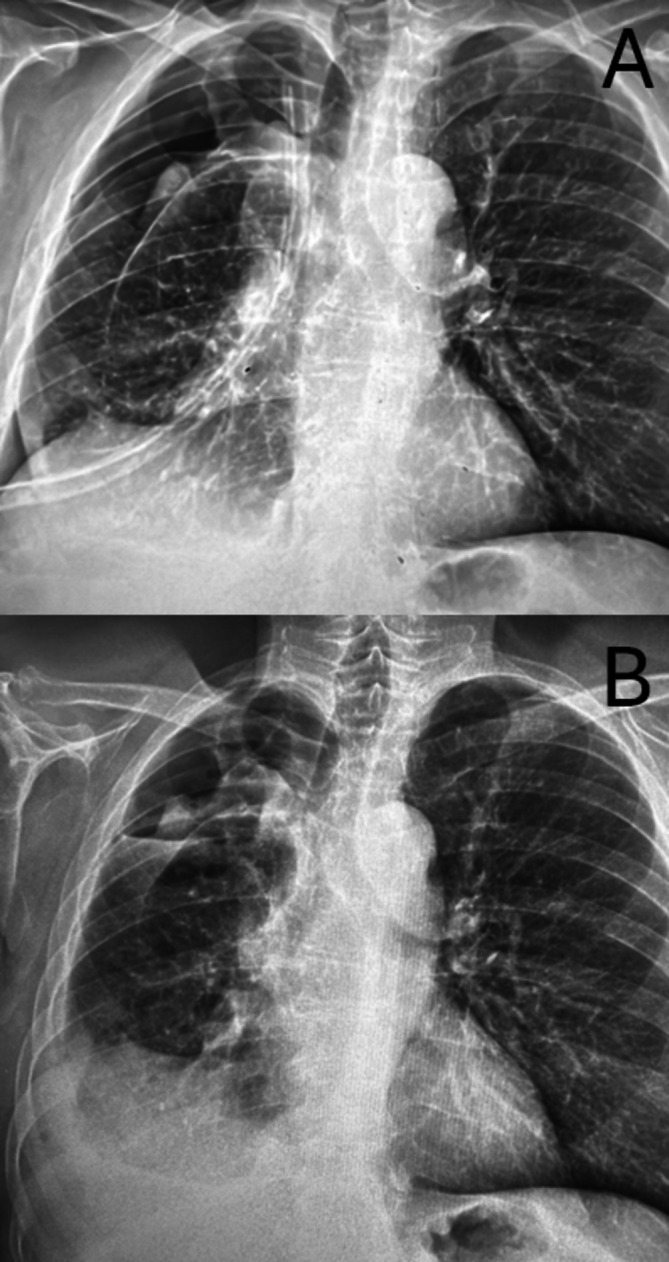
Chest X‐ray (A) at readmission showed incomplete expansion of the lung despite the presence of chest drainage (A). Chest X‐ray (B) at discharge showed the lung re‐expansion with pleural flap and apical extra‐pleural space. No chest drainage was present.

A 67‐year‐old man with a previous medical history of diabetes and emphysema underwent thoracoscopic right upper lobectomy for management of lung adenocarcinoma (T2bN0M0). Interlobar fissures were divided by stapler and then the staple lines were covered by cellulose patch. The water submersion test showed no intraoperative air leaks, and a chest drainage was left in the pleural cavity. However, air leaks occurred on post‐operative day (POD) 2; a digital pleural drainage device with a low suction pressure was connected to a chest tube to provide a continuous and objective measure of air leaks. During the postoperative course, a progressive and significant reduction of airleaks was registered, thus no attempts were performed to treat them. On POD 11, the measurement of flow rate was below the threshold of 70 mL/min, and the patient was discharged home with a Heimlich valve. However, the air leaks did not spontaneously resolve, and the patient was re‐admitted to our unit 45 days later. Chest X‐ray (Figure 1A) and computed tomography scan showed residual apical space in the absence of other lesions, and bronchoscopy demonstrated a healed upper bronchus stump without evidence of defect.

**FIGURE 2:**
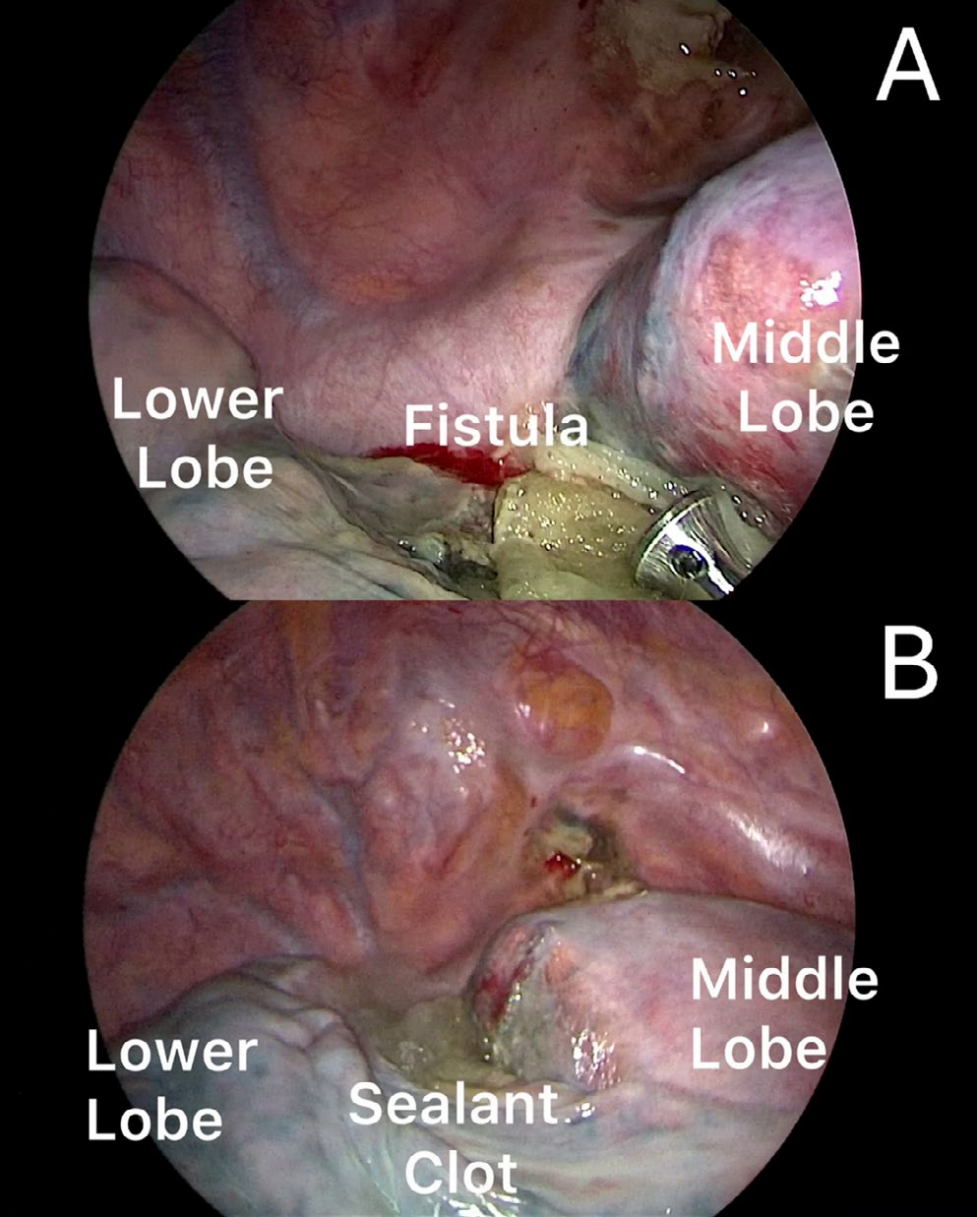
A fistula was found in the apical segment of the lower lobe at the level of the staple line where the patch was detached (A). The fistula was closed by sealant application (B).

**FIGURE 3:**
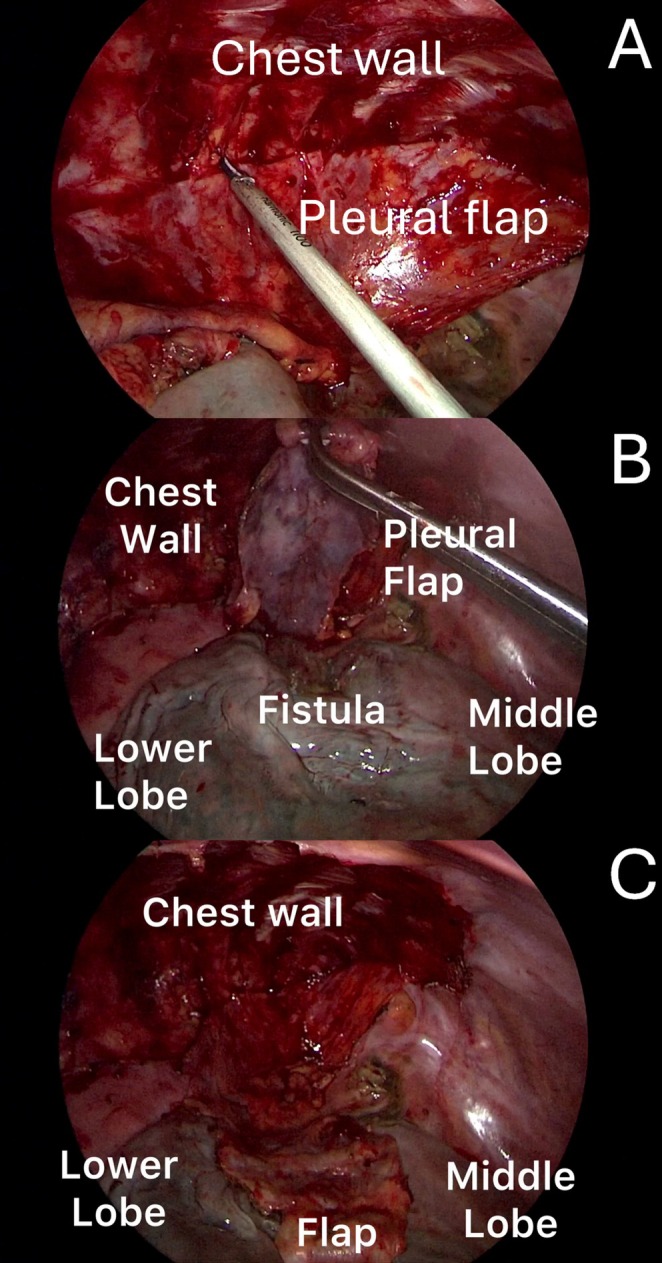
The pleural flap was peeled from the chest wall using the Harmonic device (A). When it was sufficiently mobilized (B), the flap covered the fistula and was fixed to the lung by sealant (C).

The digital pleural drainage device showed an increase in flow rate from 70 mL/min at discharge to 350 mL/min upon re‐admission, suggesting a worsening of the underlying lung disease cause of air leaks. Thus, the patient was scheduled for surgical treatment. A biportal thoracoscopy was performed using the previous surgical incisions. An alveolar fistula was found in the apical segment of the lower lobe at the level of the staple line where the patch was detached (Figure 2A). The lower lobe and middle lobe were tenaciously adhered to mediastinal pleura and hilar structures. Thus, the fistula was closed by the application of sealant (Figure 2B). Then a pleural flap was fashioned to reinforce the closure. The hook diathermy incised the parietal pleura and raised a flap that was peeled down from the chest wall using the Harmonic device (Figure 3A). The parietal pleura was taken down completely posteriorly, anteriorly, and superiorly. When the flap was sufficiently mobilized (Figure 3B), it covered the fistula and was fixed to the lung by sealant (Figure 3C). The lung was gently reinflated, and the pleural flap appeared to be tightly stuck to the lung tissue. Then, the water submersion test showed no intraoperative air leaks. Talc pleurodesis completed the operation and one chest tube was left in the pleural cavity. Video S1 summarized the procedure.

Post‐operative course was unremarkable, and air‐leaks were monitored by digital pleural system device. The flow rate was below the threshold of 50 mL/min until POD 3, then air‐leaks spontaneously ceased. On POD 5, the drainage was clamped, and a chest x‐ray was performed 24 h later. As no change was observed, the drainage was subsequently removed and the patient discharged on POD 7. Chest x‐ray (Figure 1B) showed the pleural flap and the lung re‐expansion with a minimal residual apical space. Two months follow‐up showed no recurrence of air leaks.

The authors obtained signed authorization from the patient as consent to publish the information disclosed in the study.

The clinical data were available on request due to privacy.

## 3 Discussion

PAL is the most common complication after lung resection [1, 2, 3]. It is associated with prolonged hospitalization, increased health costs, and worse outcomes. Upper resection, incomplete or fused fissures, emphysema, and underlying lung disease were identified as significant risk factors for developing PALs in most series [4, 5, 6] and in the present. Current guidelines recommend surgical treatment for the management of PAL while endoscopic procedures or pleurodesis are indicated in patients deemed unfit for surgery [7, 8]. Several surgical strategies have been reported over the years for the closure of APF. However, the best treatment is still debated as each of these procedures presents specific limitations, and the choice of treatment depends on the characteristics of the defect and patient anatomy.

This case presented all reported risk factors for the development of post operative PAL as an upper resection was performed, the interlobar fissures were fused, and the lung parenchyma was emphysematous. Despite the staple line being reinforced by cellulose patch, pleural pressure together with higher mechanical forces in the proximity of the staple lines detached the patch and caused the rupture of lung parenchyma. The lung was not fully expanded on chest X‐ray performed at the re‐admission; thus we decided against bed pleurodesis. Due to good clinical condition and the lack of severe co‐morbidities, the patient was scheduled for early surgical treatment based on current recommendations [7]. However, all previous strategies above reported were unfeasible or at high risk of failure. The stapler resection of the fistula was unfeasible as the lung presented tenacious hilar adhesions making the mobilization at high risk of vascular lesion, while the direct closure with stitch could predispose to further fistula due to the underlying disease. In similar cases, other authors [9, 10, 11] successfully closed the fistula by application of sealants or of pleural tent, but in our case these strategies were at high risk of failure due to the localization of the lesion. To overcome this limitation, we closed the fistula by application of sealant. Then, to avoid the clot dislocation and to reinforce the closure, the pleural flap covered the repaired defect, and it was then fixed by sealant.

For the success of the procedure, we recommend sufficiently mobilizing the pleural flap to cover the fistula and the adjacent normal lung. We used a harmonic device for this procedure; it allowed us to peel the parietal pleura from the chest wall and to secure hemostasis. Furthermore, at the end of the procedure, the lung should be gently re‐expanded under vision to identify any dislocation of the pleural tent and sealant clot. Our procedure did not present any specific contraindication. In theory, it was not indicated in patients with coagulopathy for the risk of bleeding from the chest wall or in those undergoing previous thoracic procedures (i.e., patients undergoing previous pleurodesis, decortication) as the presence of adhesions made difficult the preparation of the pleural tenttechnically difficult.

In conclusion, our strategy could turn out to be useful for surgeons when standard procedures for the management of APF were unfeasible or difficult to perform. Obviously, our impression should be validated by future large studies in a prospective manner.

## Author Contributions

**Alfonso Fiorelli:** conceptualization, write paper, supervision. **Noemi Maria Giorgiano:** data collection. **Angela Iovine:** data collection. **Antonella Tamburrino:** data collection. **Gaetana Messina:** write paper.

## Disclosure

The authors have nothing to report.

## Consent

An informed written consent was obtained by the patient for the publication of the study.

## Conflicts of Interest

The authors declare no conflicts of interest.

## Supporting information


**Video S1:** The video edits the main steps of the procedure as the identification of fistula; the closure of defect with glue; the fashion of pleural tend; and the fixation of pleural tend over the fistula by glue to resolve air leaks.

## Data Availability

The data that support the findings of this study are available on request from the corresponding author. The data are not publicly available due to privacy or ethical restrictions.
